# Thank you reviewers- CytoJournal 2006

**DOI:** 10.1186/1742-6413-3-2

**Published:** 2006-02-03

**Authors:** Vinod B Shidham, Barbara F Atkinson

**Affiliations:** 1 Dept of Pathology, Medical College of Wisconsin, Milwaukee, WI, USA; 2 Exective Vice Chancellor & Executive Dean, University of Kansas School of Medicine, Kansas City, KS, USA

## Abstract

The Editorial Board of CytoJournal devotes significant efforts, time, and resources to review numerous manuscripts. As it is impossible to include all the experts on the editorial board, there is always a need for additional peers who are requested to join periodically to act as 'academic editors' and reviewers. We take this opportunity to thank all the reviewers and academic editors who offered their time and efforts by participating in peer-review process for CytoJournal manuscripts. We request their continued enthusiasm to support this important academic exercise.

According to the 'close review' policy of CytoJournal, the peer-reviewers are not identified in the individual manuscripts and articles. But the 'academic editors', who conduct and complete the peer-review process by appointing reviewers are acknowledged in the articles [1,1-9]. However, all the submitted manuscripts are not accepted for publication. The academic editors, reviewing such manuscripts, can not be thanked through the published articles. As a token of appreciation and thanks, CytoJournal would publish periodically the list of all the academic editors and reviewers involved in the peer-review process of all the manuscripts submitted to CytoJournal irrespective of their publication status.

On behalf of CytoJournal's editorial body and Cytopathology-Foundation Inc, we take this opportunity to specifically thank all reviewers and academic editors listed in the Table 1. Their time and efforts for reviewing the manuscript and improving the quality of the published articles in CytoJournal [10-26] is commendable. The quality of peer-review is extremely important to continue and enhance the high standards of CytoJournal.

**Table 1:** CytoJournal reviewers and academic editors* (2006).

Andrea Abati, MD
Atilla Aridogan, MD
Zubair Baloch, MD, PhD
Michael W. Beaty, MD
Behnaz Behmaram, MD
Joel S. Bentz, M.D
George Birdsong, MD
Francisco Xavier BFX Bosch, MD
David Chieng M.D., M.B.A.
Douglas Clark, MD
Harvey Cramer, M.D
Richard M. DeMay, MD
William C. Faquin, MD
Patricia Fetsch, M.T. (ASCP)
Kim R. Geisinger, MD
Carolyn Ernst Grotkowski, M.D
Murat Golden, MD
Prabodh Gupta, MD
Karen Gustafson, MD
Michael Henry, MD
Rana Shafiq Hoda, MD
Sherif Ibrahim MD, PhD
Reena Jain, MD
Darshana N. Jhala, MD
Rani Kanthan, MD
Kusum Kapila, MD, FAMS, FIAC, FRCPath
Ruth L. Katz M.D
Amer Khiyami, MD
Orly Korat, MD.
Lester J. Layfield, MD
Gladwyn Leiman MBBCh, FIAC, FRCPath
Sanjay Logani, M.D.
Natalia Markelova, MD
Dennis R.McCoy, JD
George K. Nagy, MD
Sonya Naryshkin, MD., F.I.A.C.
Aziza Nassar, MD
Ritu Nayar, MD
Santo V. Nicosia, MD
Thaira Oweity, MD
Vishnu Reddy, MD
Andrew A. Renshaw, MD
David L. Rimm, MD, PhD
Joram Sawady, MD
Malcolm Schinstine, PhD, MD,
SuzanneSelvaggi, MD
Mark ESherman, MD
Mark S.Sidoti, Esq
Jan F. Silverman, MD
Paul E. Wakely, MD
Alfredo AFV Feria Velasco, MD
Nancy Young, MD

*We apologize in advance if any of the reviewers that participated in CytoJournal review process priore to Jan 2006 have not been acknowledged in this list. Please contact cytojournal@mcw.edu and communicate the lapse, we will include the missing reviewer in the next list. We thank you in advance for your understanding.

We also thank all the authors that submitted the manuscripts to CytoJournal and supported the 'open access' initiative in cytopathology. 'Open access' extends numerous benefits to the authors including the retention of their copyrights to the published material [27].

We invite all the experts in cytopathology and related areas to join the ever increasing demand of good reviewers. High standard of peer-review process of CytoJournal is the topmost agenda at periodic 'reviewer's retreats'. Existing editorial board members and potential core reviewers could participate at such retreats [28]. It is a great opportunity to be involved actively for the benefits of 'open access' initiative in cytopathology. Open access offers limitless potential advantages to our profession and general public by providing free access to the scientific literature generated by us as academia by collective *pro bono *hard work. To join as a core reviewer of CytoJournal, please e-mail the details by copy pasting the brief form (Table 2) in the body of your e-mail and also attaching a copy of your CV to cytojournal@mcw.edu. A list of all the core reviewers of CytoJournal is planned to be displayed on the web page in the future.

**Table 2:** Send details to join CytoJournal core reviewer panel*.

Fill this form after copy-pasting it as body of e-mail.
Send to Cytojournal@mcw.edu with your CV.
Name:
E-mail address:
Qualifications:
Affiliation:
Mailing Address:
Phone:
Fax:
Area of interest (to review cytology articles for CytoJournal):
Memberships: ▪ USCAP, ▪ ASC, ▪ Other__________
Any other comment(s):

*In the future a consolidated list of all core reviewers will be displayed on the web page.

Sincerely,  Vinod B. Shidham, MD, FRCPath, FIAC  Executive editor and co-editor-in-chief Barbara F. Atkinson, MD Co-editor-in-chief

## References

[B1] Regina Meara, Vishnu Reddy, Juan Pablo Arnoletti, Darshana Jhala, Shyam Varadarajulu, Nirag Jhala (2006). Diagnosis of Hairy Cell Leukemia by Endoscopic Ultrasound Guided Fine Needle Aspiration: A case report and review of recent literature. CytoJournal.

[B2] Gupta N, Nijhawan R, Srinivasan R, Rajwanshi A, Dutta P, Bhansaliy A, Sharma S Fine needle aspiration cytology of primary thyroid lymphoma: a report of ten cases. Cytojournal.

[B3] Garbar C, Mascaux C, Fontaine V Efficiency of an inexpensive liquid-based cytology performed by cytocentrifugations: a comparative study using the histology as reference standard. Cytojournal.

[B4] Hernandez O, Oweity T, Ibrahim S Links Is an increase in CD4/CD8 T-cell ratio in lymph node fine needle aspiration helpful for diagnosing Hodgkin lymphoma? A study of 85 lymph node FNAs with increased CD4/CD8 ratio. Cytojournal.

[B5] Nguyen GK, Lee MW, Ginsberg J, Wragg T, Bilodeau D Fine-needle aspiration of the thyroid: an overview. Cytojournal.

[B6] Arain S, Walts AE, Thomas P, Bose S The Anal Pap Smear: Cytomorphology of squamous intraepithelial lesions. Cytojournal.

[B7] Payne MM, Rader AE, McCarthy DM, Rodgers WH Merkel cell carcinoma in a malignant pleural effusion: case report. Cytojournal.

[B8] Gatter KM The big problem of the missing cytology slides. Cytojournal.

[B9] Shidham VB, Komorowski R, Macias V, Kaul S, Dawson G, Dzwierzynski WW Optimization of an immunostaining protocol for the rapid intraoperative evaluation of melanoma sentinel lymph node imprint smears with the 'MCW melanoma cocktail'. Cytojournal.

[B10] Gupta N, Srinivasan R, Nijhawan R, Dhaliwal LK Primary fallopian tubal transitional cell carcinoma with exfoliation of malignant cells in cervical Pap smear. Cytojournal.

[B11] Schultz S, Pinsky GS, Wu NC, Chamberlain MC, Rodrigo AS, Martin SE Fine needle aspiration diagnosis of extracranial glioblastoma multiforme: Case report and review of the literature. Cytojournal.

[B12] Bui M, Khalbuss WE Primary small cell neuroendocrine carcinoma of the urinary bladder with coexisting high-grade urothelial carcinoma: a case report and a review of the literature. Cytojournal.

[B13] Garza-Guajardo R, Mendez-Olvera N, Flores-Gutierrez JP, Hernandez-Martinez S, Candanosa-Mc Cann M, Ancer-Rodriguez J, Barboza-Quintana O Fine needle aspiration biopsy diagnosis of metastatic neoplasms of the breast. A three-case report. Cytojournal.

[B14] Bilic M, Welsh CT, Rumboldt Z, Hoda RS Disseminated primary diffuse leptomeningeal gliomatosis: a case report with liquid based and conventional smear cytology. Cytojournal.

[B15] Beaty MW, Geisinger KR Hodgkin lymphoma: flow me?. Cytojournal.

[B16] Pantanowitz L, Cao QJ, Goulart RA, Otis CN Diagnostic utility of p16 immunocytochemistry for Trichomonas in urine cytology. Cytojournal.

[B17] Chivukula M, Dincer HE, Biller JA, Krouwer HG, Simon G, Shidham V FNAB cytology of extra-cranial metastasis of glioblastoma multiforme may resemble a lung primary: a diagnostic pitfall. Cytojournal.

[B18] Bean SM, Eloubeidi MA, Eltoum IA, Cerfolio RJ, Jhala DN Preoperative diagnosis of a mediastinal granular cell tumor by EUS-FNA: a case report and review of the literature. Cytojournal.

[B19] Wee A Fine needle aspiration biopsy of the liver: Algorithmic approach and current issues in the diagnosis of hepatocellular carcinoma. Cytojournal.

[B20] Granja NM, Begnami MD, Bortolan J, Filho AL, Schmitt FC Desmoplastic small round cell tumour: Cytological and immunocytochemical features. Cytojournal.

[B21] Leiman G Anal screening cytology. Cytojournal.

[B22] Shidham VB, Cafaro AF, Atkinson BF CytoJournal's move to fund Open Access. Cytojournal.

[B23] Rosenthal DL, Remembering George L, Wied MD February 7, 1921-July 25, 2004. Cytojournal.

[B24] Fadare O, Mariappan MR, Hileeto D, Zieske AW, Kim JH, Ocal IT Desmoplastic Infantile Ganglioglioma: cytologic findings and differential diagnosis on aspiration material. Cytojournal.

[B25] Gupta N, Kumar V, Nijhawan R, Srinivasan R, Rajwanshi A FNAC of Bacillus- Calmette- Guerin lymphadenitis masquerading as Langerhans cell histiocytosis. Cytojournal.

[B26] Nayar R, Solomon D Second edition of 'The Bethesda System for reporting cervical cytology' – atlas, website, and Bethesda interobserver reproducibility project. Cytojournal.

[B27] Shidham VB, Cafaro A, Atkinson BF CytoJournal joins 'open access' philosophy. Cytojournal.

[B28] Retreat for new & existing CytoJournal^® ^reviewers- Feb 18, 7:10 am to 8:30 am. During USCAP Annual Meeting, Venue: Auburn Room, Hyatt Regency. http://www.cytopathology-foundation.org/page11.html.

